# Milling overrides cultivar, leavening agent and baking mode on chemical and rheological traits and sensory perception of durum wheat breads

**DOI:** 10.1038/s41598-017-14113-5

**Published:** 2017-10-19

**Authors:** Donatella Bianca Maria Ficco, Sergio Saia, Romina Beleggia, Mariagiovanna Fragasso, Valentina Giovanniello, Pasquale De Vita

**Affiliations:** Council for Agricultural Research and Economics - Research Centre for Cereal and Industrial Crops (CREA-CI), S.S. 673 km 25.200, 71122 Foggia, Italy

## Abstract

Smell and aroma are important determinants of consumer acceptance, so gaining deeper insight into bread smell and aroma perception is a research goal. Sixteen combinations of four variables were investigated, to evaluate the contributions of bread chemical and rheological properties and volatile organic compounds (VOCs) towards sensory acceptability of breads: genotypes (landrace *vs*. modern); types of flour (wholemeal *vs*. semolina); leavening agents (brewing yeast *vs*. sourdough starter); and baking modes (gas-fired *vs*. wood-fired). Milling had the greatest impact over the other treatments for the rheological and chemical properties, including for VOCs, with great impact on the sensory traits of the flours and breads. The processing phases had great impact on smell and aroma, as defined through formation of alcohols, aldehydes, terpenes, and other compounds (e.g., ethylbenzene, 2-pentylfuran, methoxyphenyl oxime). Leavening agent had great impact on sensory perception, although breads from the sourdough starter were perceived as with lower taste and colour than the brewing yeast. Baking mode had no relevant role on sensory perception. These data strongly undermine the belief of a ‘better product’ that is frequently attributed to old genotypes *versus* modern cultivars, and indicate that the milling and the bread-making processes determine the quality of the end product.

## Introduction

Durum wheat [(*Triticum turgidum* (L.) ssp. *durum* (Desf.)] is the main ingredient of pasta, couscous, bulgur and some breads in Mediterranen areas^1^. In Italy, approximately one quarter of the durum wheat is consumed in several forms of bread^2^. Such breads are very heterogeneous, obtained through different methods, although they share some common characteristics. Most regional breads are indeed produced through traditional methods, which include the use of flours (e.g., wholemeal, semolina) from specific durum wheat genotypes, and the long-term use of natural locally refreshed sourdoughs for leavening. This frequently results in breads with a yellowish colour, a characteristic taste, smell and aroma, a fine crumb structure, and a prolonged shelf-life, all of which give these breads high appeal to consumers.

While the consumption of bread is generally declining in developed countries, significant groups of consumers appear to prefer speciality breads prepared with wholemeal/semolina or from old wheat genotypes^3^. This trend seems to depend on the perceived quality of these breads by consumers, who attributes them particular sensory and nutritional attributes, marketing *stimuli* (e.g., traceability), and hedonistic content (e.g., storytelling)^3–5^. Some consumers, for instance, perceive higher nutritional and functional value and better sensory properties of wholemeal bread from old genotypes prepared using traditional/ancient methods (such as cooking in wood oven), compared to bread from modern cultivars using industrial milling processes, and leavened with brewing yeast^6,7^.

Indeed, durum wheat cultivars, milling technique, leavening agent, and baking mode can influence the main technological, nutritional, functional and sensory properties of bread^8,9^. Numerous studies have highlighted the importance of the flour, the gluten composition, and the genotype on bread traits^2,10^. Wholemeal/semolina flours have different composition and performances during bread baking, which results in different bread types^11^. When bran is added, the loaf volume decreases^12^, but the nutritional value increases^11^. Furthermore, some studies have demonstrated the effectiveness of sourdough leavening to improve volume, shelf-life, and nutritional value of such cereal products^13^. In addition, ingredient formulation, dough leavening, and baking conditions can significantly influence bread smell and aroma (see Cho and Peterson^14^; and Pico *et al*.^15^). More than 540 volatile organic compounds (VOCs) have been reported from bread, although few actually contribute to flavour, smell and aroma^14–17^. Each VOC can be perceived at the different concentration that define its individual activity threshold^18^.

Several studies have applied descriptive sensory analysis to define the consumer perception of different breads^19^, but most studied as single treatments^4,8,20,21^. No previous reports are available regarding the interaction of the bread-making variables (i.e., durum wheat genotype, milling process, leavening agent and baking) on the chemical, rheological and sensory traits of the final bread product.

For genotype, protein content and gluten strenght are two of the most important determinants of pasta and bread making, as they strongly influence textural traits, loaf volume, and appearance^8^. As consumers’ acceptance is not solely dependent on loaf volume, there is a need to investigate which variable has the major role in the sensory perception of durum wheat bread.

In the present study, we combined chemical and rheological analyses, and VOCs emission from the flours to the corresponding breads, to study the impact on sensory perception. In particular, we compared the full combination of 16 different experimental breads obtained with old *vs*. modern durum wheat cultivar, milled as semolina *vs*. wholemeal flour, leavened with brewing yeast *vs*. sourdough starter, and baked in gas-fired *vs*. wood-fired oven.

## Results

### Flours characteristics and VOCs

Genotype (*G*) and milling (*M*) strongly affected the chemical and rheological parameters and VOCs emitted by flours, whereas fewer effects were seen for the *G* × *M* interaction (Table 1). In particular, the modern cultivar ‘Sfinge’ showed lower protein, total and soluble fibre contents, and higher gluten index than the old cultivar ‘Dauno III’. Ash content and semolina colour (measured as yellow index) were similar between genotypes.

**Table 1:** Chemical and rheological properties of the durum wheats, and VOCs from their flours.

Durum wheat genotype (G)	Milling product (M)^a^	Chemical properties (%)										Rheological properties				VOCs from milling product (ng g^−1^)							
		Protein		Ash		Fibre						Gluten index (%)		Yellow index		Total^b^		Alcohols		Aldehydes		Terpenes	
						Total		Insoluble		Soluble													
Old ‘Dauno III’	Wholemeal	15.2 ± 0.04		1.7 ± 0.01		13.8 ± 0.29		10.3 ± 0.20		3.5 ± 0.08		1.0 ± 0.04		19.4 ± 0.06		5.7 ± 0.17		1.3 ± 0.14		2.9 ± 0.22		0.6 ± 0.13	
	Semolina	14.1 ± 0.01		0.9 ± 0.01		5.8 ± 0.22		3.4 ± 0.14		2.4 ± 0.08		3.2 ± 0.16		25.0 ± 0.30		4.1 ± 0.08		1.3 ± 0.07		1.5 ± 0.17		1.3 ± 0.10	
Modern ‘Sfinge’	Wholemeal	14.6 ± 0.02		1.6 ± 0.01		12.4 ± 0.31		10.8 ± 0.27		1.6 ± 0.04		55.5 ± 2.04		19.6 ± 0.01		8.5 ± 0.61		2.9 ± 0.03		3.8 ± 0.54		1.2 ± 0.04	
	Semolina	13.4 ± 0.02		0.9 ± 0.01		5.0 ± 0.18		3.5 ± 0.10		1.5 ± 0.08		90.7 ± 0.18		25.3 ± 0.12		7.5 ± 0.01		3.7 ± 0.06		2.1 ± 0.04		1.6 ± 0.02	
**Analysis of variance (General linear mixed model)**		***F***	***p***	***F***	***p***	***F***	***p***	***F***	***p***	***F***	***p***	***F***	***p***	***F***	***p***	***F***	***p***	***F***	***p***	***F***	***p***	***F***	***p***
*G*		**828**	<**0.001**	8.0	0.066	**226.1**	**0.001**	**16.3**	**0.027**	**3249**	<**0.001**	**3040**	**0.012**	0.98	0.426	**99.8**	<**0.001**	**561**	<**0.001**	5.0	0.089	**40.7**	<**0.001**
*M*		**2739**	<**0.001**	**12325**	<**0.001**	**11711**	<**0.001**	**6303**	<**0.001**	**529**	<**0.001**	**206**	**0.005**	**1213**	**0.001**	**17.6**	**0.006**	**26.2**	**0.002**	**34.3**	**0.004**	**75.1**	<**0.001**
*G* × *M*		**10.4**	**0.049**	18.0	**0.024**	**19.16**	**0.022**	5.8	0.095	**441**	<**0.001**	**265**	**0.004**	0.05	0.849	0.55	0.488	**26.7**	**0.002**	0.65	0.466	2.31	0.180

^**a**^Milling products indicate flours;

^**b**^Total VOCs include alcohols, aldehydes, terpenes and other compounds

Data are means ± standard error. Treatments and interaction at *p* < 5% are shown in bold.

For the milling process, wholemeal showed higher protein, total fibre and ash content, and lower gluten and yellow indexes compared to semolina. In addition, ‘Dauno III’ wholemeal had 1.1% more soluble fibre than its semolina. No differences between the two flours for ‘Sfinge’ were found. The VOCs released by flours grouped in VOC classes (i.e., alcohols, aldehydes, terpenes) were ∼1.5–2.0-fold higher in ‘Sfinge’ than ‘Dauno III’, and in wholemeal than semolina, with few *G* × *M* interactions. In particular, a *G* × *M* interaction for the VOC classes released by the milling product was seen only for alcohols, which did not differ between flours for ‘Dauno III’, but were higher for the ‘Sfinge’ semolina than wholemeal.

### Bread texture and chemical properties

**Figure 1:**
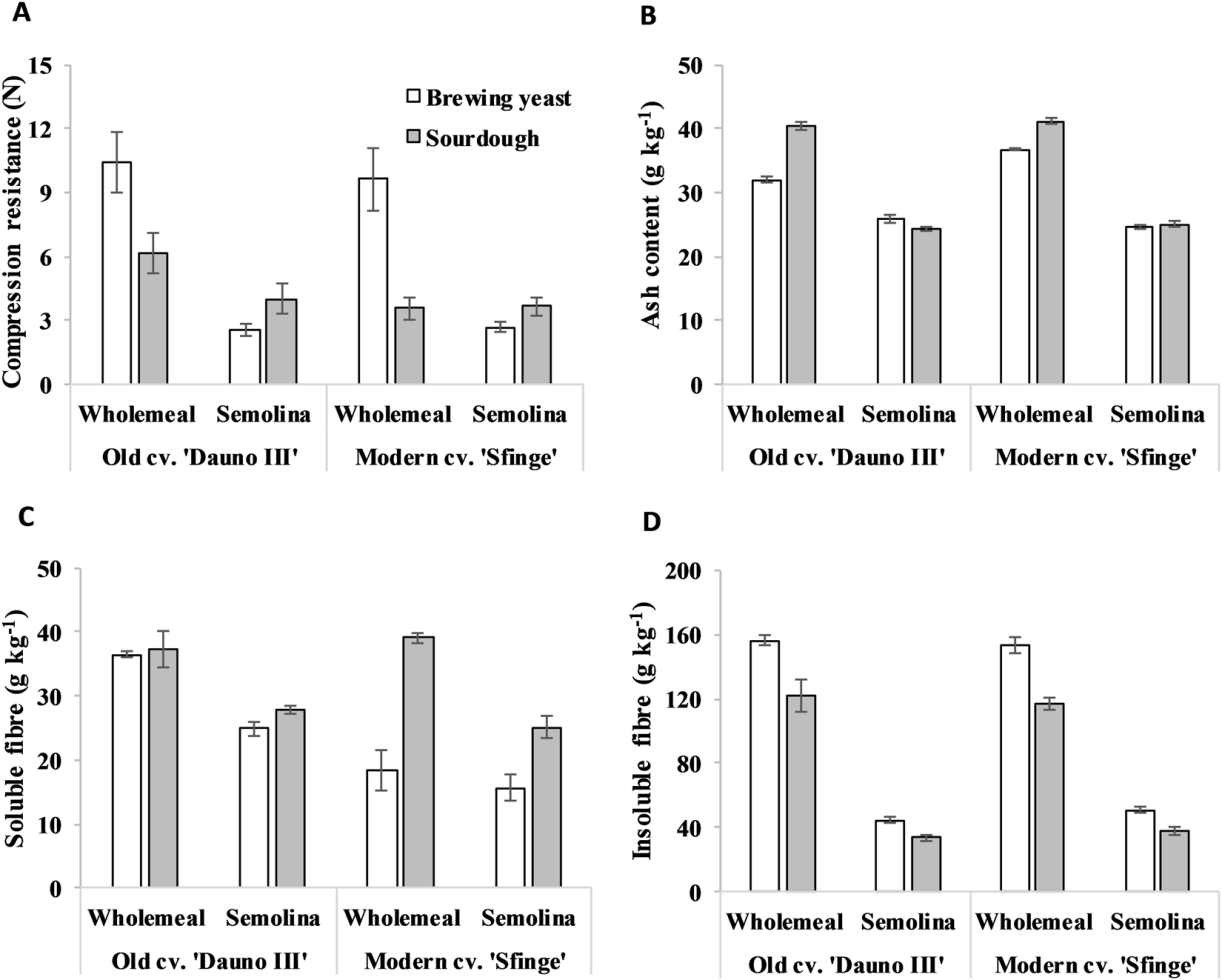
Effects of genotype, milling product and leavening agent on chemical performance and texture of durum wheat breads. (**A**) Compression test. (**B**) Ash content. (**C**) Soluble fibre. (**D**) Insoluble fibre. Genotype, old genotype ‘Dauno III’ *versus* modern genotype ‘Sfinge’; milling product, wholemeal *versus* semolina; leavening agent, brewing yeast (open columns) *versus* sourdough (shaded columns).

The treatments applied strongly influenced the chemical and textural bread parameters, that mostly varied by milling, leavening (*L*) and their interactions with each other and with the other treatments. Genotype had few effect (Table 2). Second (3-way) and third order (4-way) interactions were for magnitude and direction of the effects. For these reasons, the *G* × *M* × *L* interaction was chosen to build figures as it more completely depicted the effects of the treatments on the variables (Fig. 1).

**Table 2:** Results of the analysis of variance (General linear mixed model) for the chemical and rheological properties, and the VOCs from the breads under study. *G*, durum wheat genotype (old, ‘Dauno III’; modern, ‘Sfinge’); *M*, milling product, i.e. flours (wholemeal; semolina); *L*, leavening agent (brewing yeast; sourdough); *B*, baking mode (gas-fired oven; wood-fired oven).

factors	Analysis of variance result																			
	Ash		Fibre						Compression resistance		VOCs									
			Insoluble		Soluble		Total				Total^a^		Alcohols		Aldehydes		Ketones		Terpenes	
	F	*p*	F	*p*	F	*p*	F	*p*	F	*p*	F	*p*	F	*p*	F	*p*	F	*p*	F	*p*
*G*	27.5	*0.120*	0.2	*0.750*	31.7	*0.112*	23.4	*0.130*	6.1	*0.245*	2.3	*0.271*	1.5	*0.345*	4.9	*0.158*	2.7	*0.245*	**84.2**	***0.012***
*M*	**2990.5**	<***0.001***	**5216.8**	<***0.001***	**57.4**	<***0.001***	**6117.8**	<***0.001***	**131.5**	<***0.001***	**195.1**	<***0.001***	**83.9**	<***0.001***	**196.8**	<***0.001***	**11.6**	***0.002***	**41.0**	<***0.001***
*G* × *M*	**45.0**	<***0.001***	**12.1**	***0.004***	0.7	*0.411*	**17.8**	***0.001***	**4.8**	***0.046***	**74.0**	<***0.001***	**65.8**	<***0.001***	**49.7**	<***0.001***	**64.0**	<***0.001***	**26.8**	<***0.001***
*L*	**162.3**	<***0.001***	**327.3**	<***0.001***	**45.5**	<***0.001***	**132.5**	<***0.001***	**28.8**	<***0.001***	**158.4**	<***0.001***	**220.3**	<***0.001***	**27.1**	<***0.001***	**106.3**	<***0.001***	**239.0**	<***0.001***
*G* × *L*	3.9	*0.067*	0.6	*0.438*	**27.7**	<***0.001***	**17.1**	***0.001***	2.4	*0.143*	**21.0**	<***0.001***	**8.3**	***0.008***	**16.4**	<***0.001***	**27.8**	<***0.001***	**372.3**	<***0.001***
*M* × *L*	**227.3**	<***0.001***	**79.7**	<***0.001***	3.4	*0.087*	**50.0**	<***0.001***	**75.3**	<***0.001***	**97.6**	<***0.001***	**58.5**	<***0.001***	**121.0**	<***0.001***	**94.8**	<***0.001***	1.9	*0.177*
*G* × *M* × *L*	**43.6**	<***0.001***	0.0	*0.989*	**7.0**	***0.019***	**6.0**	***0.028***	0.9	*0.368*	**8.2**	***0.008***	**18.7**	<***0.001***	0.3	*0.591*	**9.3**	***0.005***	1.2	*0.282*
*B*	0.6	*0.444*	**49.3**	<***0.001***	2.2	*0.164*	**30.7**	<***0.001***	0.0	*0.848*	**80.9**	<***0.001***	**40.9**	<***0.001***	**91.4**	<***0.001***	**25.1**	<***0.001***	0.0	*0.952*
*G* × *B*	2.6	*0.131*	0.4	*0.536*	0.1	*0.820*	0.2	*0.689*	1.3	*0.271*	**35.1**	<***0.001***	**38.5**	<***0.001***	**11.1**	***0.002***	**66.6**	<***0.001***	0.7	*0.411*
*M* × *B*	2.9	*0.109*	**22.7**	<***0.001***	2.6	*0.127*	**10.1**	***0.007***	0.1	*0.780*	**151.4**	<***0.001***	**119.7**	<***0.001***	**97.9**	<***0.001***	**160.4**	<***0.001***	**10.4**	***0.003***
*G* × *M* × *B*	**7.2**	***0.018***	3.5	*0.083*	1.0	*0.335*	**7.7**	***0.015***	0.0	*0.942*	2.2	*0.148*	1.1	*0.310*	1.0	*0.335*	**9.8**	***0.004***	1.0	*0.334*
*L* × *B*	2.6	*0.131*	1.3	*0.269*	0.6	*0.445*	0.2	*0.696*	0.6	*0.465*	3.2	*0.087*	**6.0**	***0.021***	0.0	*0.916*	1.6	*0.223*	0.5	*0.499*
*G* × *L* × *B*	2.2	*0.157*	1.4	*0.260*	0.1	*0.811*	0.9	*0.370*	1.1	*0.313*	1.8	*0.194*	4.0	*0.056*	0.2	*0.649*	**30.1**	<***0.001***	0.2	*0.636*
*M* × *L* × *B*	0.0	*0.936*	1.8	*0.206*	0.7	*0.426*	0.3	*0.599*	0.1	*0.835*	0.3	*0.617*	0.3	*0.596*	0.0	*0.952*	**16.1**	<***0.001***	2.8	*0.105*
*G* × *M* × *L* × *B*	0.3	*0.577*	**10.6**	***0.006***	0.5	*0.484*	**6.4**	***0.024***	3.8	*0.070*	0.8	*0.391*	0.5	*0.490*	2.0	*0.174*	0.1	*0.749*	**7.2**	***0.012***

^a^Total VOCs include alcohols, aldehydes, terpenes and other compounds

Data are means ± standard error. Treatments and interaction at *p* lower than 5% are shown in bold.

For both of the genotypes, the highest firmness values were seen for the wholemeal breads, which appeared to be due to the presence of bran (Fig. 1A). Moreover, the sourdough wholemeal breads were softer (compression resistance, −52%) than wholemeal brewing-yeast-leavened breads for both genotypes. On the contrary, the sourdough semolina breads showed slightly higher compression resistance than the brewing yeast semolina bread.

For both cultivars, ash content differed mostly according to milling, with higher values in wholemeal than semolina breads (Fig. 1B). In addition, in the wholemeal breads of both cultivars, sourdough showed 6.4 g kg^**−1**^ more ash than the brewing yeast breads.

Soluble fibre of both cultivars was 9.5 g kg^**−1**^ more abundant in the wholemeal than the semolina breads (Fig. 1C). For ‘Dauno III’, there were no differences in soluble fibre content between the leavening agents, whereas for ‘Sfinge’, sourdough showed 20.6 g kg^**−1**^ and 9.4 g kg^**−1**^ more soluble fibre than brewing yeast breads made with the wholemeal and semolina, respectively.

Insoluble fibre of wholemeal was 95 g kg^**−1**^ more than semolina breads, and that of brewing yeast was 24 g kg^**−1**^ more than sourdough, with few effects of genotype and interactions (Fig. 1D).

### VOC classes in breads

Differences among treatments for total VOCs, alcohols and aldehydes were similar (Supplementary Figure S1A–C). In particular, the genotype had little effect on these traits. For wholemeal breads, those that were wood fired showed 97% more total VOCs than those that were gas fired, with no differences between leavening agents. Similar data were seen for alcohols and aldehydes (+89%, + 131%, for wood-fired and gas-fired, respectively). For semolina breads, brewing yeast showed 302%, 293% and 661% more total VOCs, alcohols and aldehydes, respectively, than sourdough, with no differences between baking modes. Differences among treatments for ketones released matched those of total VOCs (Supplementary Figure S1D), but for semolina breads, those gas-fired released more ketones than wood-fired. Terpenes of wholemeal ‘Dauno III’ were higher than semolina ‘Dauno III’ breads (Supplementary Figure S1E), with no effects of leavening agent and baking. For ‘Sfinge’, there were more terpenes for breads leavened with the brewing yeast than sourdough, with no effects of milling and baking. Finally, differences in other compounds released from breads (i.e., ethylbenzene, 2-pentylfuran, methoxyphenyl oxime) were similar to those of the aldehydes (Supplementary Figure S1F).

### VOC profiles in breads

**Figure 2:**
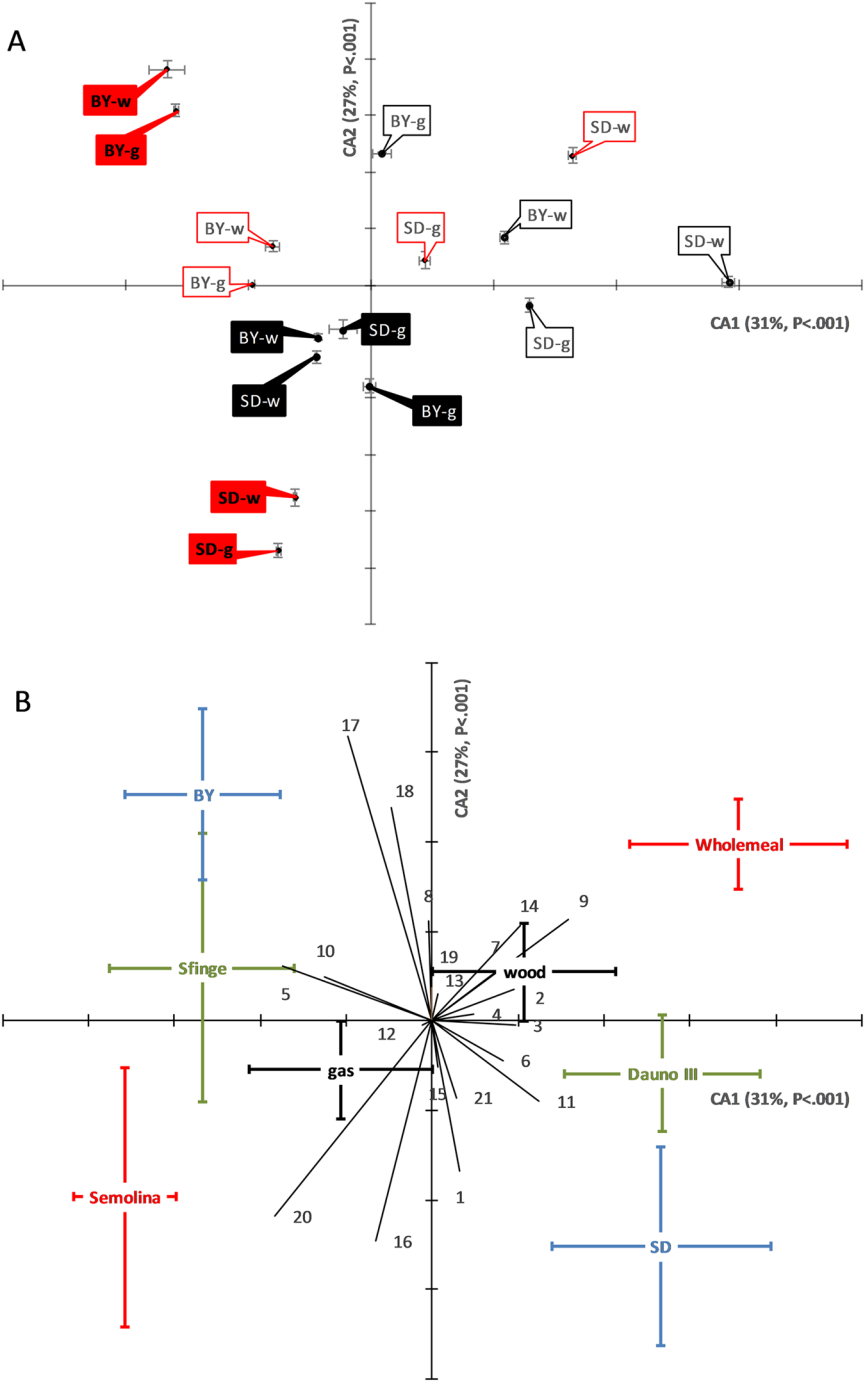
Canonical Discriminant Analysis using standardised data for the single VOCs from the durum wheat bread analysis. (**A**) Distribution of centroid means (±S.E.; n = 3) on the canonical axes (CA) 1 and 2 for each bread. Black labels, old cv. ‘Dauno’; red labels, modern cv. ‘Sfinge’; open labels, wholemeal; closed labels, semolina. Leavening agents were brewing yeast (BY) or sourdough (SD). Baking was gas-fired (g) or wood-fire (w) oven. The percentage of the total variance explained by each canonical axis is shown in parentheses. (**B**) Vectors of the variables. 1, 1-methylbutanol; 2, hexanol; 3, 2-ethyl-1-hexanol; 4, octanol; 5, phenylethyl alcohol; 6, heptanal; 7, nonanal; 8, benzaldehyde; 9, decanal; 10, limonene; 11, 6-methyl-5-hepten-2-one; 12, *trans*-3-octen-2-one; 13, methoxyphenyl oxime; 14, 2-pentylfuran; 15, ethylbenzene; 16, 2,3-butandiol; 17, furanmethanol; 18, heptanol; 19, 1-octen-3-ol; 20, nonanol; 21, 3-furaldehyde/ furfural. Positioning of the mean of each treatment (centroid ±S.E.; n = 24) is shown. Green, mean of genotypes; red, milling; blue, leavening agent; black, baking mode. Note that CDA vectors do not represent perpendicular directions through the space of the original variables. The unit of measure is the same for both axes.

Changes in VOC profile were studied using Canonical Discriminant Analysis (CDA) run on standardised data of single VOCs. CDA separated milling, genotype and leavening agent on Can1 (Fig. 2A; 31% variance explained, *p* < 0.001) and milling and leavening agent on Can2 (27% variance explained, *p* < 0.001). On average, there was separation of wholemeal from semolina bread, which was mainly dependent on nonanal, hexanol and heptanal on the Can1 (Fig. 2B), and 1-methylbutanol, 2,3-butandiol, furanmethanol, heptanol and benzaldehyde on Can2. Decanal, 2-pentylfuran and nonanol were equally distributed along the two axes. The genotypes under study mainly differed in terms of 2-ethyl-1-hexanol, phenylethyl alcohol and limonene, which mainly affected Can1. Also, leavening agents were separated along Can1 due to phenylethyl alcohol and limonene, and along Can2 due to heptanol, furanmethanol and 6-methyl-5-hepten-2-one. Heptanol, octanol, nonanol, heptanal, methoxyphenyl oxime, *trans*-3-octen-2-one and 2-pentylfuran also contributed to Can3 (13% variance explained, *p* < 0.001, data not shown), on which milling and baking treatments were separated.

**Figure 3:**
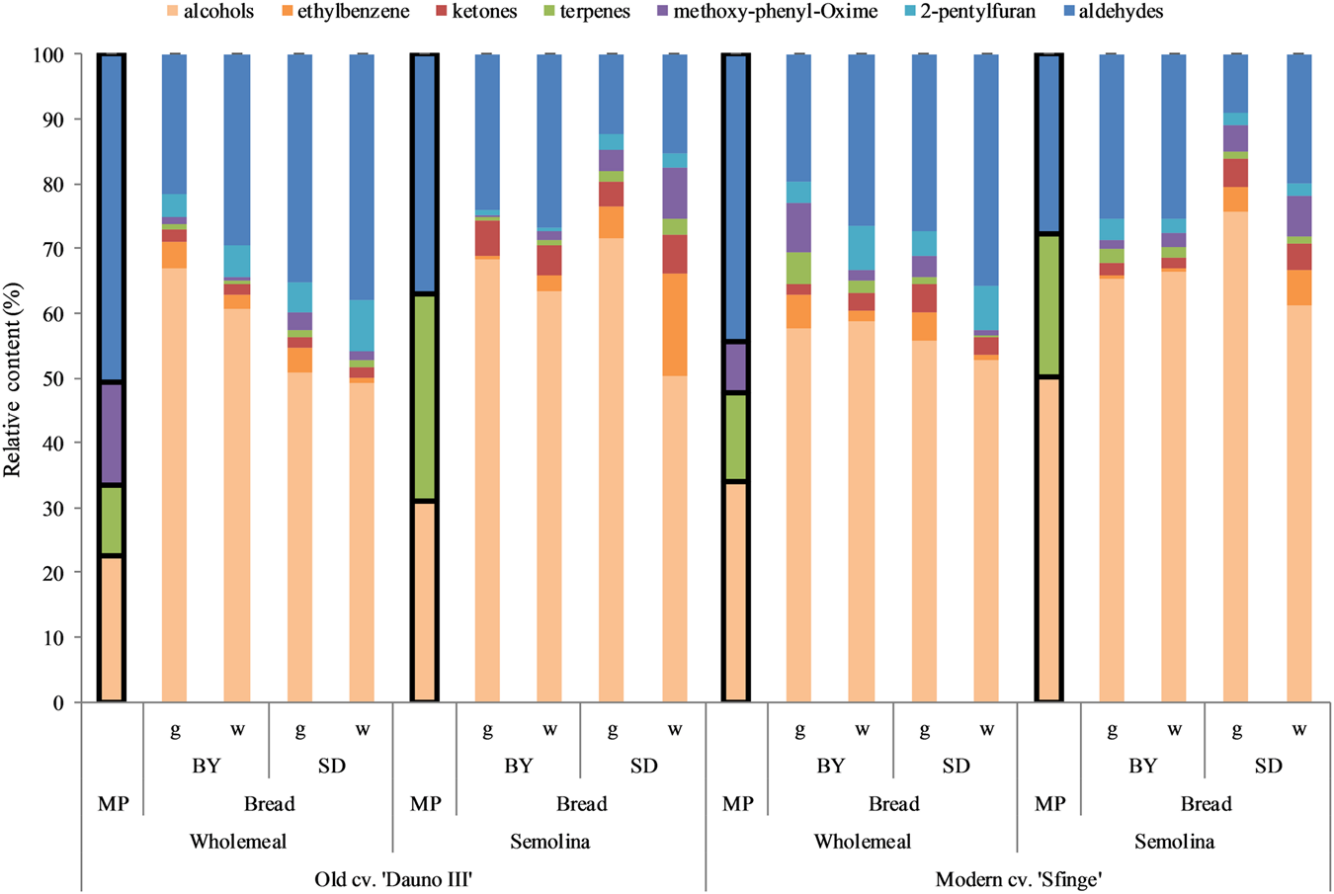
Relative contents of each class of compounds in the breads and flours. MP, milling products, i.e flours (black-bordered columns), for either wholemeal or semolina flour (as indicated). Leavening agents were brewing yeast (BY) or sourdough (SD). Baking was gas-fired (g) or wood-fire (w) oven.

For the contribution of each VOC class to the total VOCs released, the effect of the bread-making process was clearly stronger than genotype and milling (Fig. 3; and please find the *F-statistics* in Supplementary Table S1). In particular, VOCs profile of each bread compared to the respective milling product showed that bread-making process resulted in an increase in alcohol:total VOCs ratio, and a reduction in the ratios of aldehydes and terpenes to total VOCs. Moreover, newly formed compounds, such as ketones, 2-pentylfuran, ethylbenzene and methoxy-phenyl-oxime, were found in each bread, irrespective of the milling product used to bake them.

### Bread sensory attributes and the importance of each treatment on consumer perception

CDA run with non-standardised data of bread sensory properties separated breads along Can1 according to milling (60% variance explained, *p* < 0.001) and along Can2 according to leavening agent (22% variance explained, *p* < 0.001), whereas no separation occurred along either Can1 or Can2 for genotype and baking (Supplementary Figure S2). Separation along Can1 mainly depended on alveolation and smell, perceived as ‘better’ in wholemeal than semolina breads, and on taste, colour and crumb firmness, recognized as ‘better’ in semolina than wholemeal breads. Separation along Can2 was mainly due to the contribution of appearance and alveolation, the latter of which was perceived as ‘better’ for sourdough than brewing yeast breads, and taste, which, on the contrary, was perceived as ‘better’ for brewing yeast than sourdough breads (Supplementary Table S2 and Supplementary Figure S3).

**Figure 4:**
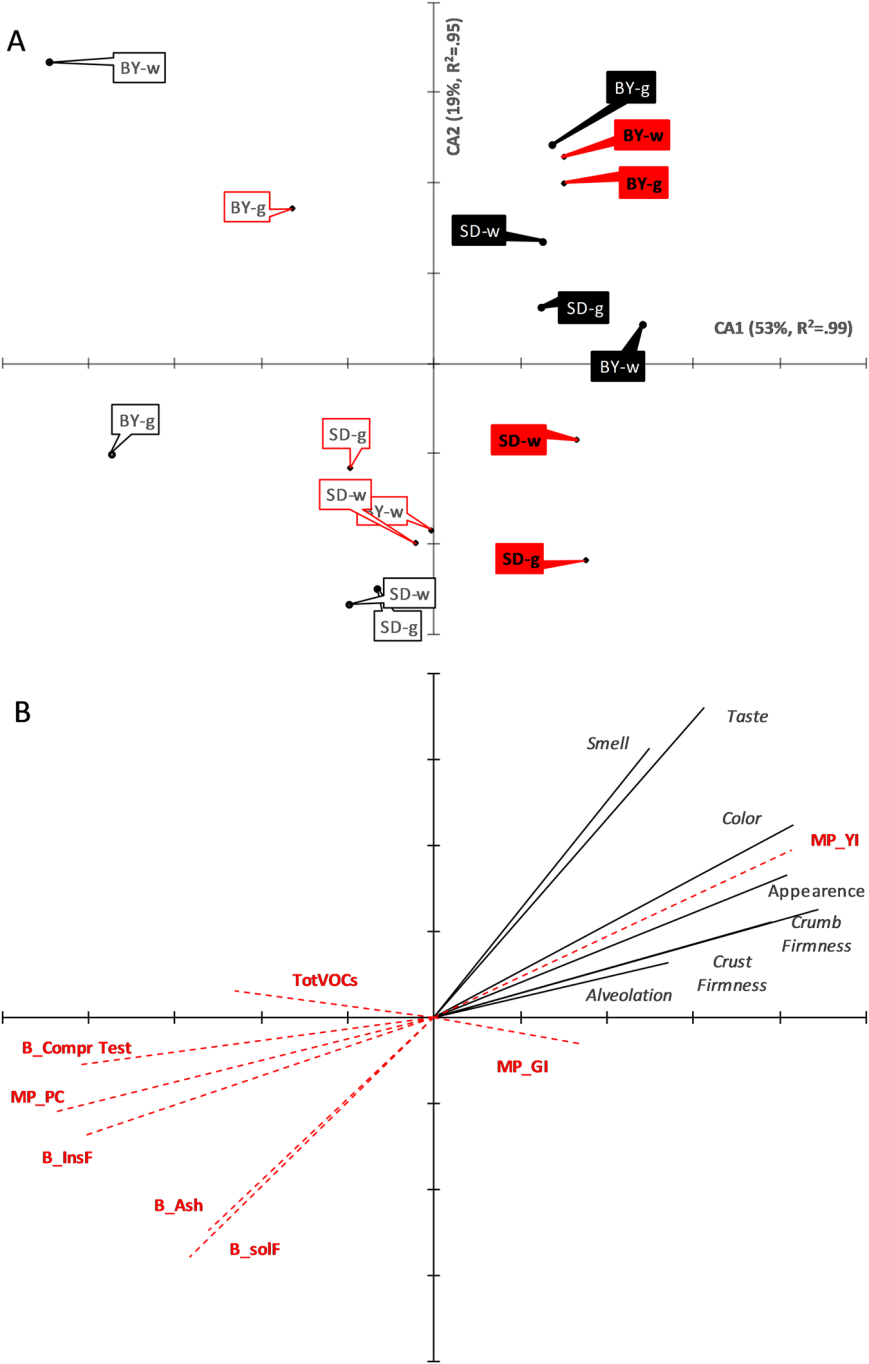
Ordination triplot of the canonical canonical correspondence analysis (CCA) of bread panel test outcomes weighted basing on bread and flours traits. Ordination triplot for CA1 vs. CA2 of the breads (panel A) calculated based on the canonical variables calculated using non-standardised panel test outcomes (continuous black lines) weighted based on non-correlated, standardised, bread and milling products, i.e flours, traits (hatched-pointed red lines) (panel B). See Materials and methods section for the rationale of standardization procedure. Units of measure for Axis 1 and Axis 2 are the same (0.5 in panel A, and 0.2 in panel B). Percentage of total variance explained by each canonical axis is shown in parentheses along with regression coefficient between panel test axis ordination and traits ordination. Each symbol represents the treatment mean on CA1 and CA2. Vectors (‘VAR’ groups, with names in italics) intersecting at (0,0) represent the original variables. Black labels are for old cv. Dauno; red labels are for modern cv. Sfinge; open labels are for wholemeal; closed labels for semolina breads. Leavening agent were brewing yeast [BY] or sourdough [SD]. Baking was gas fire [g] or wood fire [w]). The contribution of the following bread and milling product chemical and reological traits (‘WITH’ groups, in red and bold names) to the panel test outcomes variability is shown: total VOCs (TotVOCs); ashes, insoluble and soluble fibre content, and compression resistance of the bread (B_Ashes, B_InsF, B_solF, and B_Compr Test, respectively); protein content, gluten index and yellow index of the milling product, i.e. flours (MP_PC, MP_GI, and MP_YI, respectively). Breads that are close to each other are similar in panel test traits corrected for the effects of bread and milling product traits. Breads that share a common VAR vector positively co-vary in panel test traits. In a similar way, similar directions of specific VAR with specific WITH vectors indicate an association between panel test traits and bread and milling product chemical and rheological traits.

**Figure 5:**
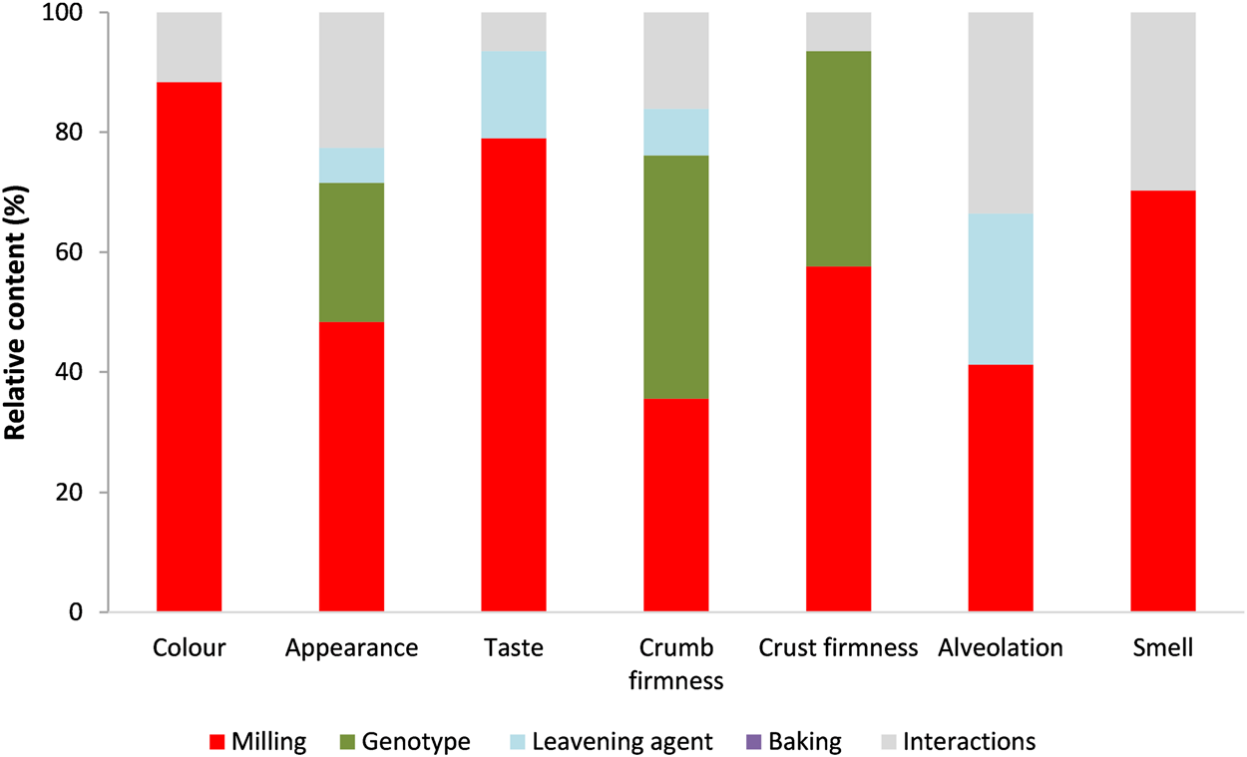
Contribution of each treatment and sum of interactions among the treatments for the total variance explained by each mixed model. For details, see text.

Distribution of breads for panel test outcomes weighted by bread and flours traits (Fig. 4, panel A) was similar to those found in the corresponding CDA (Supplementary Figure S2) for the milling product effect but not for the leavening agent, which appeared less important than genotype. Weighting of the panel test scores by the bread and flours traits (Fig. 4, panel B) clearly showed that perception of colour and appearance of the bread was related, as expected, to yellow index, and in turn to taste and smell. Gluten index was slightly, but positively associated with taste. Many bread and milling product traits negatively corrected perception scores, especially protein content, insoluble fibre and compression test, and to a minor extent soluble fibre and ash. Among the treatments, the largest effects seen on the bread sensory attributes were due to the milling procedures, especially for colour, smell and taste and similarly but at a minor extent for crust and crumb firmness, appearance and alveolation (Fig. 5). Genotype was important only in crust and crumb firmness and appearance. Leavening agent (<14%) and whole of interactions (<20%,) had little influence, and baking had no relevant role on sensory properties.

## Discussion

Recently, a distorted vision of wheat-based food has been ‘hyped’ in the mass media, leading to unsubstantiated concerns about its the safety and health implications^22^. With regard to bread and pasta, many concerns focus on modern cultivars, pointing at their gluten index as an anti-nutritional trait related to digestion problems and rise in the incidence of coeliac disease (for a detailed report see Kang *et al*.^23^,), which is however strongly controversial and likely not supported by data. In addition, adoption of modern genotypes has been indicated as a main reason for loss of the sensory properties of pasta and breads.

We analysed the effect of genotype, milling, leavening agent, and baking modes on rheological traits, VOC emission, and sensory perception of the bread.

As expected, the landrace ‘Dauno III’ showed more protein and lower gluten index than the moden cultivar ‘Sfinge’, as a common effect of breeding^24^. Other differences between old and modern genotypes have also been seen for fibre and mineral contents, gluten quality, growth habit, and morphological and physiological features^24–29^, which can directly or indirectly influence the characteristics of flours and end products. Instead, colour, a parameter well discriminated by the consumers, did not show differences between genotypes. Analyzing flours, the dietary fibre of the wholemeal durum wheat in the present study is in the range reported by others^30^. The present data show a higher content of water-soluble fibre in ‘Dauno III’ than ‘Sfinge’, which confirms the differences shown previously between ancient and modern bread wheat genotypes by Ormoli *et al*.^25^ (and references therein).

In terms of VOCs composition, genotype appeared relatively important, as previously reported by others^14,15^, but its contribution to bread flavour was minor compared to other treatments, especially milling. According to others^31^, low-refined flour could have a role in the production of lactic acid compared to refined-flour, during the sourdough leavening, and this can affect VOCs composition, although their effect was limited if compared to processing. Indeed, many identified compounds responsible for bread smell are formed from the leavening agent metabolism^9,18,32^, are strongly influenced by its strains^32–34^, and enhanced upon the baking^18^.

For the processing, increased crumb firmness for wholemeal breads compared to semolina breads has been reported previously^12,35^, as related to bran concentration. Leavening agent showed an opposite effect on firmness of semolina and wholemeal bread probably due to the different physico-chemical composition of the matrices. Sourdough improved texture, volume and shelf-life in high fiber wheat bread^36,37^, but its influence on bread firmness is not completely elucidated since other changes occuring in the starch–protein matrix affect this trait^37^. So, further studies are needed to clarify the effect of leavening on bread properties.

The higher ash content in wholemeal sourdough than brewing yeast breads likely depended on the effect of the starter on the final humidity of the breads, as also suggested by the opposite result for firmness, indirectly affecting volatility of the more polar VOCs. Also, high ash and thus mineral content in wholemeal flours (see Table 1) can stimulate the microflora in the sourdough^38^, and some strains of lactic acid bacteria (LAB) can divert a fraction of the fermentable sugars on biosynthesis of exopolysaccharides, which have positive effects on the final porosity and resistance to retrogradation^39^. Finally, wholemeal sourdough leavening has been shown to enhance formation of tiny bubbles and phytate hydrolysis, which favour mineral solubility^39,40^, compared to brewing yeast leavening.

The different behaviour of soluble fibre in the sourdough (see Fig. 1) compared to brewing yeast breads was likely due to the interaction of starch and non-starch polysaccharides with the structure and internal bonding of gluten, that imply disulfide bonds, which could have affected the bioavailability of nutrients for LAB and habitability for yeast during leavening. Moreover, the increase in soluble fibre content observed here, in particular for ‘Sfinge’, and according to Johansson *et al*.^41^, could be due to partial conversion of insoluble to soluble fibre, which was more likely in sourdough, as a result of either the fibre fermentation^42^ or other fractions differing in the two genotypes used.

Also, differences in other chemical and sensory properties for the wholemeal bread might depend on LAB strains in the starter, as shown for semolina breads^32^, that can largely contribute to the evolution of smell and aroma compounds^32,33^.

Very little is known on the interaction among different VOCs (and their relative concentrations) on the flavour perceived, especially when sulphur-rich proteins and sugars occurs (such as in durum wheat)^43,44^. We identified limonene and many important non-terpene compounds (e.g., methylbutanol, phenylethyl alcohol, 2,3-butandiol, furanmethanol, heptanal), that strongly differed among breads, as reported in previous studies^18^. This reflected in the panel test discrimination by the milling product effect, which, however, depended on other traits rather than smell and aroma (but see Supplementary Figure S2).

Kneading, leavening and baking have been indicated as critical steps for bread smell and aroma^15,16^. We show that genotype, leavening agent and baking contribution for smell and aroma perception were low, whereas they were significant for VOCs composition. This discrepancy might be due to the high levels of VOCs derivatives that can affect smell and aroma. For example, phenylethyl alcohol has up to 43 flavouring derivatives^44^. Thus, a correspondance of bread smell to its VOC composition should rely on the equivalent smell and aroma type and odour activity. This limitation occurred in the present study even though the method and conditions used (head-space, solid phase, microextraction at ambient temperature collection, which impairs non-microbial assisted interconversion of compounds to those with higher activation energy) allowed the extraction of VOCs representative of bread smell and aroma^17^. Quílez *et al*.^45^ showed a relationship between chemical traits (including VOCs) and sensory perception using heated (to 50 °C) bread samples. In addition, we also found that some compounds (i.e., ketones, 2-pentylfuran, ethylbenzene, methoxyphenyl oxime) were found in bread but not in flour VOCs. These compounds likely resulted from thermal degradation of lipids^46^, the occurrence of which in the grain also depend on the genotype^20,47^. Vita *et al*.^48^ used heated samples of grain and wholemeal (to 60 °C) and reported similar amounts of compounds as the present study, which indicates that some changes in the aromatic profile of wheat can occur at the grinding phase.

We saw here that the bread-making process (i.e., kneading, the leavening process, baking) was mostly responsible for changes in VOC classes from flours to breads, and that leavening agent had minor effects on these changes. Thus, as the bread-making effect on VOCs changes from flour to bread was similar across genotypes and flours, we hypothesise that kneading and the leavening process, with minor role of the agent, have strong roles in VOCs production and sensory properties. Similarly, Czerny and Schieberle^49^ reported that LAB leavening did not generate new aldehydes, but instead altered the relative concentrations of those that were present in both wholemeal and white flour.

Similar to the effects of grinding^48^, the increased aldehydes, presence of new ketones, and decreased alcohols to the total VOCs in breads compared to flours might also depend on lipid oxidation, as reported for pasta^20,50^, given that lipoxygenases^51^ is active during breadmaking phases^52^. At the same time, occurrence of 2-pentylfuran and some aldehydes might have been due to the plant enzyme activity after grinding (and probably also during kneading, when the mass humidity allows for enzymatic activity)^53^, or to thermally enhanced linoleic acid autoxidation during the baking^54^.

Wood-fired baking consisted in larger amount of VOCs than gas-fired, as also shown by Bianchi *et al*.^52^. In our experiment, this effect resulted more evident in wholemeal bread, probably due to its flour composition (i.e., gluten, non-starch polysaccharides, lipids) more rich in precursors involved in the non-enzymatic browing reactions as well as to the interaction between leavening agent strains and food matrix^33^. The higher total VOCs found in brewing-yeast semolina compared to sourdough semolina breads could probably be attributed to the condition adopted. In fact, although different amounts of leavening agent (see Materials and Method) were used to leaven dough at similar extent and rate, leavening time was the same and probably not enough in sourdough semolina bread to the release of a variety of VOCs by the LAB activity.

On the whole, the sensory evaluation revealed differences among breads mainly depending on the milling product used, and to a lesser extent on the genotype and leavening agent. Moreover, these differences were more related to the rheological properties of the flours than to the VOCs composition of both flours and breads, suggesting that texture/mouthfeel was a greater determinant of sensory properties than smell and aroma. Thus, it’s likely that the ‘better’ taste perceived in the semolina breads leavened with brewing yeast (Supplemental Material Table 2; Supplemental Material Fig. 3) depended directly on the absence of the bran, and indirectly on the other chemical and rheological traits. These data confirm those by Quílez *et al*.^45^, who indicated that consumers preferred bread with a greater degree of leavening by yeast, without secondary fermentation by LAB. However, ur-Rehman *et al*.^55^ reported contrasting data in winter wheat breads. These discrepancies migh arise from both the amount of starter used and ability of the LAB to produce exopolysaccharides^56^.

Also proteins and gluten index affected bread taste (see Fig. 4). A bread loaf of good quality is influenced by protein content and protein quality as well as by crumb properties. The last one can differ in terms of strength, alvelation, cell size and morphology. Gluten quality plays an important role in the formation and maintenance of gas bubbles. In particular, the lower protein content in Sfinge than Dauno III is counterbalanced by higher gluten strength to keep the gas bubbles in the gas cells and mantain them during bread making. The CCA showed that gluten index (Fig. 4 panel B) had a more evident and positive role in affecting bread perception, together with colour, than protein content, which was negatively linked with sensory value attribution.

Finally, the higher amounts of fibre and ash in wholemeal than semolina breads resulted in a darker color and a not always positive effect on smell for the bitterness of flour, more rich in bran fractions, as well as aromatics associated with wholegrain that has been roasted/burnt^57^. However, although the consumer acceptability of the wholemeal dark bread is limited, the beliefs in its health promoting attributes (in which the dietary fibre co-exist with other active compounds) are pointed out.

Our approach and data show that the factors studied can have solid contributions to the chemical and rheological characteristics and sensory properties of the final bread, although the interaction among these factors was of little importance in bread perception.Consideration of these findings could drive the consumer and producers’ criteria for bread choice and manufacture, respectively.

However, we show here that the effect of genotype can be masked by other treatments, in particular milling, and also probably fibre content. In addition, it has been shown that the environment can have strong effects on the production of phytochemicals for different winter wheat genotypes, and that some modern genotypes can accumulate healthy phytochemicals to an extent that is similar to that of many old genotypes^58^. Similarly, it has been shown that both ‘chemically measured’ and perceived qualities of durum wheat bread vary little according to the genotype, and that the perception of bread quality instead depends on the protein content and its quality, and soluble fibre contents rather than other traits^48,59^.

In conclusion, semolina breads were generally perceived as better than wholemeal breads, except for smell. Taste, colour and crumb firmness and appearance were the main factors that contributed to increased ‘perception of good flavour’ in the durum wheat breads. The preference for smell of wholemeal over that of semolina breads was supported partly by the VOCs composition. Leavening also strongly affected the ‘perception of good flavour’, and this mostly depended upon alveolation, which might have contributed to the differences in the VOC composition, and taste. Finally, the genotype contributed to the ‘perception of good flavour’ only to a certain extent; these effects were only clear for the appearance of the bread and crust and crumb firmness, and they were strongly overridden by the milling procedure to obtain the flour (wholemeal or semolina), which was definitely the strongest determinant of the flavour.

These data indicate milling and bread-making processes highly involved in the achievement of a bread superior quality, and strongly undermine the beliefs of ‘better product’ frequently attributed to the old genotypes, in defiance of the modern cultivars. This is especially the case here considering that we used recently harvested grain, where antioxidant and lipid compositions were unaltered. Indeed, products made with flour/semolina of old genotypes frequently undergo mild milling (which partly conserves its antioxidant properties) and are leavened with sourdough starters (which also strongly contribute to their perception).

## Methods

### Ethic statement

The present article does not report experiments on humans or use of human tissue samples. The bread used was prepared according the Italian national and EC regulations. The Sorelle Palese Bakery (Potenza, Italy), involved in the present experiment, is authorised and controlled for safety and hygiene according the Italian rules. No institutional or licensing committee approving the experiments was needed to prepare the breads and flours. All participant to the sensory analysis were made aware of potential risks. No informed consent was needed to allow anyone to participate to the sensory analysis since it is not a medical procedure nor involves non natural or harmful compounds or chemicals or drugs.

### Flours and experimental design

Two durum wheat genotypes were used: the landrace ‘Dauno III’, cultivated between 1915 and 1930, and the modern variety ‘Sfinge’, released in 2004. The grain used for bread making was produced at CREA-CI in Foggia, Italy (41°28′N, 15°32′E; 75 m a.s.l.) during growing season 2013–2014. For soil and agronomical practices applied to the plots, see De Vita *et al*.^27^. The experiments used a split-plot design replicated twice. Main treatment was durum wheat genotype (*G*): ‘Dauno III’ or ‘Sfinge’. Sub-treatments were milling (*M*): wholemeal or semolina; leavening agent (*L*): brewing yeast or sourdough; and baking mode (*B*): gas-fired or wood-fired oven.

### Milling procedures

Grain samples were tempered to 16.5% moisture, separated in two sub-samples, and milled using two different procedures. Semolina was produced bya laboratory mill (MLU 202; Bühler Brothers, Uzwill, Switzerland) fitted with three breaking and three sizing passages, and a semolina purifier. Wholemeal flour was obtained by a stone mill (MB250; Partisani, Forlì, Italy), with three fractions recovered, as flour, fine bran and bran. Fractions used for bread making were flour (particle size, <260 μm) and fine bran (particle size, 260–1060 μm), in 30:70 (w/w) ratio, and herein referred as ‘wholemeal’.

### Bread-making process

Breads were prepared using industrial bakery equipment at Sorelle Palese Bakery, and its sourdough leavening agent. Each dough was prepared with: 2,000 g durum wheat semolina or wholemeal of each genotype, 16 g compressed brewing yeast (*Saccharomyces cerevisiae*) or 80 g of 30-year-old mother sponge daily refreshed, 60 g sodium chloride, and 1600 mL tap water. Basing on previous trials, different amounts of leavening agents were used to achieve the same leavening times of the dough. Dough was obtained by mixing flour, water and salt in a mixer (Conti kneaders, Verona, Italy); then the starter was slowly added and mixed with the other ingredients at 4 rpm for 15 min, and then at 120 rpm for 5 min. The dough derived under each experimental condition was weighed (three dough pieces, each 1,000 g ± 2 g), manually rounded, and placed in a temperature-controlled proofing oven (Thermogel, Varese, Italy) at 30 °C ± 1 °C and 85% ± 1% relative humidity, and leavened for 60 min. Then, each dough was re-rounded and allowed to leaven for a further 3 h. Each uncooked, leavened bread was baked for 45 min in a preheated gas-fired oven (Europa Forni, Vicenza, Italy) at 300 °C, or for 1 h in a wood-fired oven at 350 °C that used bundles of twigs from the Lucania woodlands. In both baking modes, bread was maintained in the oven after the end of baking until oven temperature was 220 °C. Samples were cooled at room temperature for 1 h before performing each sampling. Three loaves were used for the characterisation of each experimental bakery. The breads were cut transversely in two halves and four central slices were sampled. A part of each sample was entirely crushed (crust and crumb) and used for chemical analysis.

### Chemical characterisation

Protein content (N × 5.7, dry weight, AACC method 46-13.01^60^) was determined by micro-Kjeldahl. Ash content (g kg^**−1**^) was determined by furnace incineration method. Gluten index (%, AACC method 38-12.02^60^) was determined using an automatic gluten washing apparatus (Glutomatic) followed by centrifugation. Yellow index was determined with a colorimeter (CR-200 tristimulus; Minolta Chroma Meter CR 200, Osaka, Japan) set to L*, a* and b* mode (CIE, 1986), and ‘b*’ value was used in the analysis as it represents the variation in yellow intensity. Total dietary, water-soluble and water-insoluble fibre (g kg^**−1**^, AACC Method 32–07^60^) were determined using Total Dietary Fibre kit (Megazyme). Measurements were performed in triplicate.

### Crumb texture analysis

Crumb texture analysis was performed according to Mastromatteo *et al*.^59^. Briefly, bread loaves were uniformly sliced (slice thickness, 15 mm) and crust cut off to allow crumb texture measurement. Cylindrical crumb samples (diameter, 28 mm) were cut from the centre of each loaf using a circular cutter. Compression test was carried out using a texture analyser (Z010 ZwickRoell Italia S.r.l., Genova, Italia). An insert plate fixed in the universal work platform (100 × 90 × 9 mm) and a compression die (diameter, 75 mm) were the parallel plates within which the cylindrical breadcrumb samples were placed. The force to compress the bread slices to a predetermined penetration level against a rigid back plate using a cylindrical plunger was recorded. Experimental conditions used a preload of 0.3 N, load cell of 1 kN, maximum percentage deformation of 50% (F_50%_) and a constant cross-head speed of 100 mm min^−1^.

### Analysis of volatile organic compounds

VOCs in the flours and bread samples were measured using static headspace, solid-phase micro-extraction (HS-SPME). Briefly, samples of 3 g flour or 0.5 g bread were added with 1 μL decane as internal standard, and subjected to HS-SPME using a 50/30 mm DVB/ Carboxen/ PDMS Stable-Flex fiber inserted in the headspace of a 40-mL amber vial with cap and PTFE/ Silicon septa (Supelco, Co., Bellefonte, PA, USA) for 30 min. The vials were maintained at 30 °C ± 0.1 °C in a water bath. After sampling, the device was immediately injected onto the column for gas-chromatography coupled with quadrupole-time-of-flight mass spectrometery (7200 system; Agilent Technologies), according to Beleggia *et al*.^20^. The VOCs were identified by comparison of their spectra with those of pure compounds contained in a custom library and NIST11 database. Relative concentrations of individual compounds (ng g^−1^) were determined after peak area normalization for that of the internal standard. Each analysis was performed in triplicate.

### Sensory analysis

Tasters were thirteen (four men, nine women; aged range, 28–45 years) familiar with sensory analysis of foods but non specifically trained in bread sensory attributes evaluation. The analysis was performed according to the methodology reported in ISO 6658^61^. The descriptors were chosen from those reported in other studies^32,59^, and referred to ‘smell and aroma’, taste, colour, aspect, crust and crumb consistence, and alveolation. Tasters expressed the intensity of each attribute in a 1 to 9 scale, where 1 corresponded to ‘extremely unpleasant’, 9 to ‘extremely pleasant’, and 5 represented the acceptability threshold.

### Statistical analysis

Data on chemical, rheological and sensory attributes, and VOCs were subjected to analysis of variance (Glimmix procedure; SAS/STAT 9.2) according to the experimental design, separately per product (flours or breads). Differences among means were compared by applying t-grouping at the 5% probability level to the LSMEANS.

To test the role of bread making process (in term of leavening and baking) in the contribution of each VOC class to the total VOCs (hereafter VOC partitioning), we applied a mixed model (Glimmix procedure; SAS/STAT 9.2) on the unbalanced dataset including VOC partitioning of both flours (i.e. 4 combinations of G × M) and breads (i.e. 16 combinations of G × M × L × B). Thus, we applied a Kenward-Roger (KR) approximation to the denominator degrees of freedom estimate and standard error, and an unstructured covariance matrix to take into account of the correlation between data of VOC partitioning from breads of a given G × M bread to those of the corresponding flour. In addition, since data were percentage, they were checked for normality by the Shapiro-Wilk test (proc. Univariate, SAS/STAT 9.2) and transformed to natural logarithm when needed. After transformation, normality was checked again.

To study how chemical and rheological traits in bread and milling products affect the panel test outcomes, a canonical correspondence analysis (CCA, proc Cancorr in SAS/STAT 9.2) was performed. CCA was run on non-correlated, standardised, bread and flour traits and non-standardised panel test outcomes. Standardisation of bread and flour traits was made to normalise for different unit of measures and correct for absolute variations. Standardisation of panel test variables was avoided since these variables have the same unit of measure, maximum achievable range and meaning (i.e. goodness of perception), thus standardization would have overweighed variables with lower scoring range. When 2 or more variables were correlated at |r| > 0.70, only 1 was retained for the analysis to avoid element-weighting distortion. See Giovino *et al*.^62^ for additional information of the rationale of variable choice and for an explanation of CCA procedure.

The importance of each treatment in affecting each panel attribute was calculated by partial eta-squared (*partial_ƞ* ^2^), as in Equation (1):

1 $$partial\,{\eta }^{2}=[({\rm{DFnum}}\times {\rm{F}})/({\rm{DFden}}+{\rm{DFnum}}\times {\rm{F}})\times 100]$$

where DFnum and DFden are the degrees of freedom of treatment and error, respectively, and F is the F statistic. This allowed the estimation of the contribution of each treatment to the effects of the total variance after the removal of the other treatments and interaction effects. The contribution of the *partial ƞ* ^2^ of each significant treatment is reported as percentages. The sum of the interaction (significant 2-, 3-, 4-way interactions) effects were also computed. Finally, two CDAs (Candisc procedure, SAS/STAT) were run using single VOCs or sensory attributes as vectors. Before running the CDA, data on single VOCs were standardised to a mean of 0 and a standard deviation of 1, to correct for variations in the absolute concentrations of each VOC.

## Electronic supplementary material

Supplementary material
