# Supplementary material for: Milling overrides cultivar, leavening agent and baking mode on chemical and rheological traits and sensory perception of durum wheat breads
Source: Sci Rep. 2017 Oct 19;7:13632. doi: 10.1038/s41598-017-14113-5 (PMC5648824; doi:10.1038/s41598-017-14113-5)
Supplement: Supplementary file 1 — Supplementary material [file 41598_2017_14113_MOESM1_ESM.pdf]

Supplementary Material Files

Milling overrides cultivar, leavening agent and baking mode on chemical and rheological traits and sensory perception of durum wheat breads

Donatella Bianca Maria Ficco*, Sergio Saia, Romina Beleggia, Mariagiovanna Fragasso, Valentina Giovanniello, Pasquale De Vita

Council for Agricultural Research and Economics - Research Centre for Cereal and Industrial Crops (CREA-CI), S.S. 673 km 25.200, 71122 Foggia, Italy

*Correspondence and requests for materials should be addressed to:

Donatella Bianca Maria Ficco

CREA-CI

S.S. 673 km 25.200

71122 Foggia, Italy

Tel: +39-088-1742972

Email: donatellabm.ficco@crea.gov.it

Legend.

Supplementary Material Figure S1. Volatile organic compounds (VOCs). Effects of genotype (landrace 'Dauno III', modern cultivar 'Sfinge'), milling product (wholemeal, semolina), leavening agent (brewing yeast [BY], sourdough [SD]), and baking mode (gas-fired oven [open columns], wood-fired oven [shaded columns]) on VOCs from the durum wheat breads. **(A)** Total VOCs. **(B)** Alcohols. **(C)** Aldehydes. **(D)** Ketones. **(E)** Terpenes. **(F)** Other compounds.

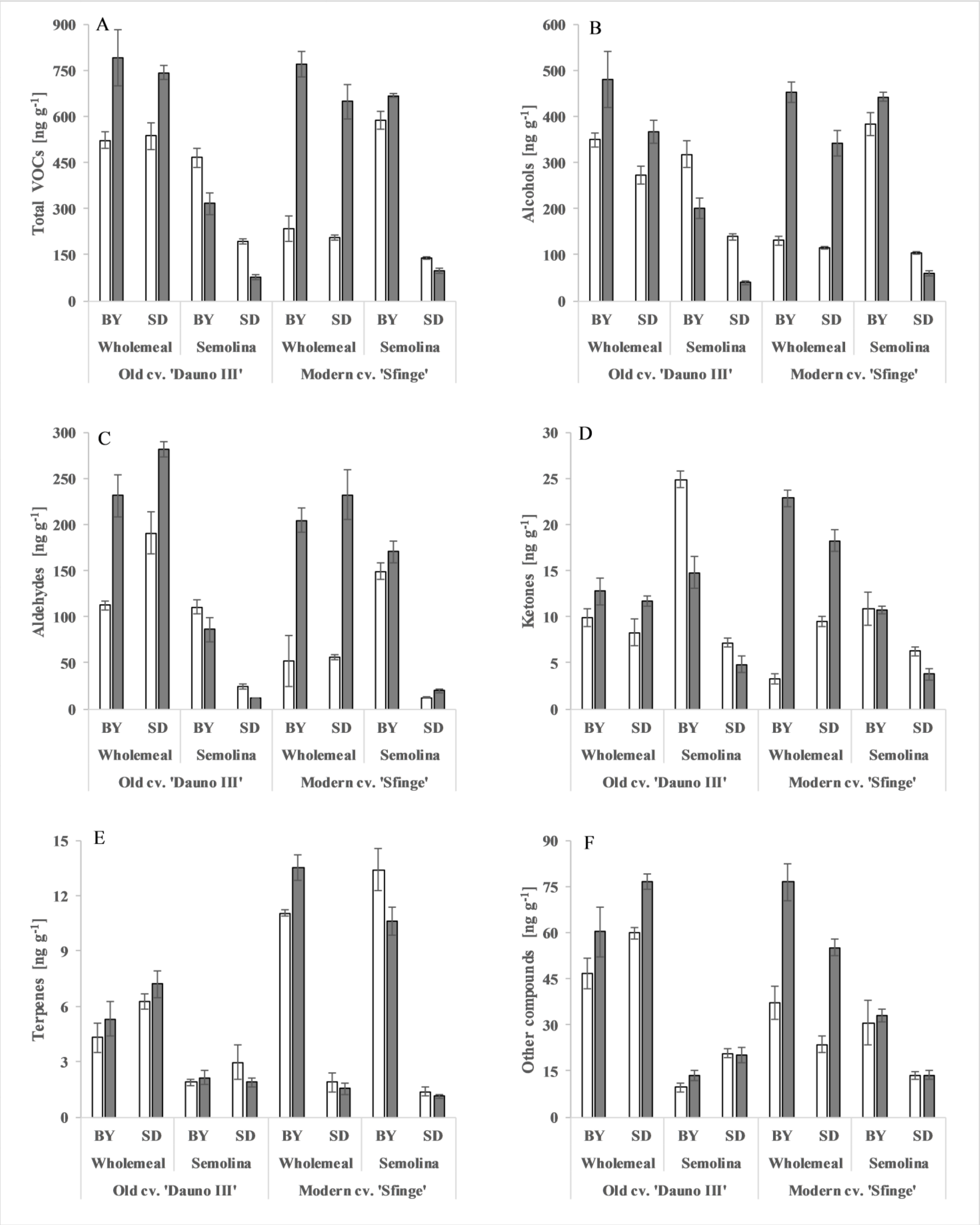

A
Total VOCs [ng g-1]
900
750
600
450
300
150
0
BY SD BY SD BY SD BY SD
Wholemeal Semolina Wholemeal Semolina
Old cv. 'Dauno III' Modern cv. 'Sfinge'
B
Alcohols [ng g-1]
600
500
400
300
200
100
0
BY SD BY SD BY SD BY SD
Wholemeal Semolina Wholemeal Semolina
Old cv. 'Dauno III' Modern cv. 'Sfinge'
C
Aldehydes [ng g-1]
300
250
200
150
100
50
0
BY SD BY SD BY SD BY SD
Wholemeal Semolina Wholemeal Semolina
Old cv. 'Dauno III' Modern cv. 'Sfinge'
D
Ketones [ng g-1]
30
25
20
15
10
5
0
BY SD BY SD BY SD BY SD
Wholemeal Semolina Wholemeal Semolina
Old cv. 'Dauno III' Modern cv. 'Sfinge'
E
Terpenes [ng g-1]
15
12
9
6
3
0
BY SD BY SD BY SD BY SD
Wholemeal Semolina Wholemeal Semolina
Old cv. 'Dauno III' Modern cv. 'Sfinge'
F
Other compounds [ng g-1]
90
75
60
45
30
15
0
BY SD BY SD BY SD BY SD
Wholemeal Semolina Wholemeal Semolina
Old cv. 'Dauno III' Modern cv. 'Sfinge'

Legend.

Supplementary Material Figure S2. Canonical Discriminant Analysis using standardised data for the panel test scores for the durum wheat breads. Each point is the centroid mean across panelists (±S.E., n = 13) on the canonical axes (CA) 1 and 2. Circles, old cv. 'Dauno III' (DA); diamonds, modern cv. 'Sfinge' (SF); open symbols, gas-fired breads (g); closed symbols, wood-fired breads (w). Groups: black lines, leavened with brewing yeast; red lines, leavened with sourdough; solid lines, prepared with wholemeal flour; dashed lines, prepared with semolina. The percentages of the total variance explained by each canonical axis are shown in parentheses. In total, two CAs were significant and explained 82.0% of the total variance. Black lines starting from '0;0' represent the vectors of each quality perception determinant. Note that the CDA vectors do not represent perpendicular directions through the space of the original variables. The unit of measure is the same for both axes.

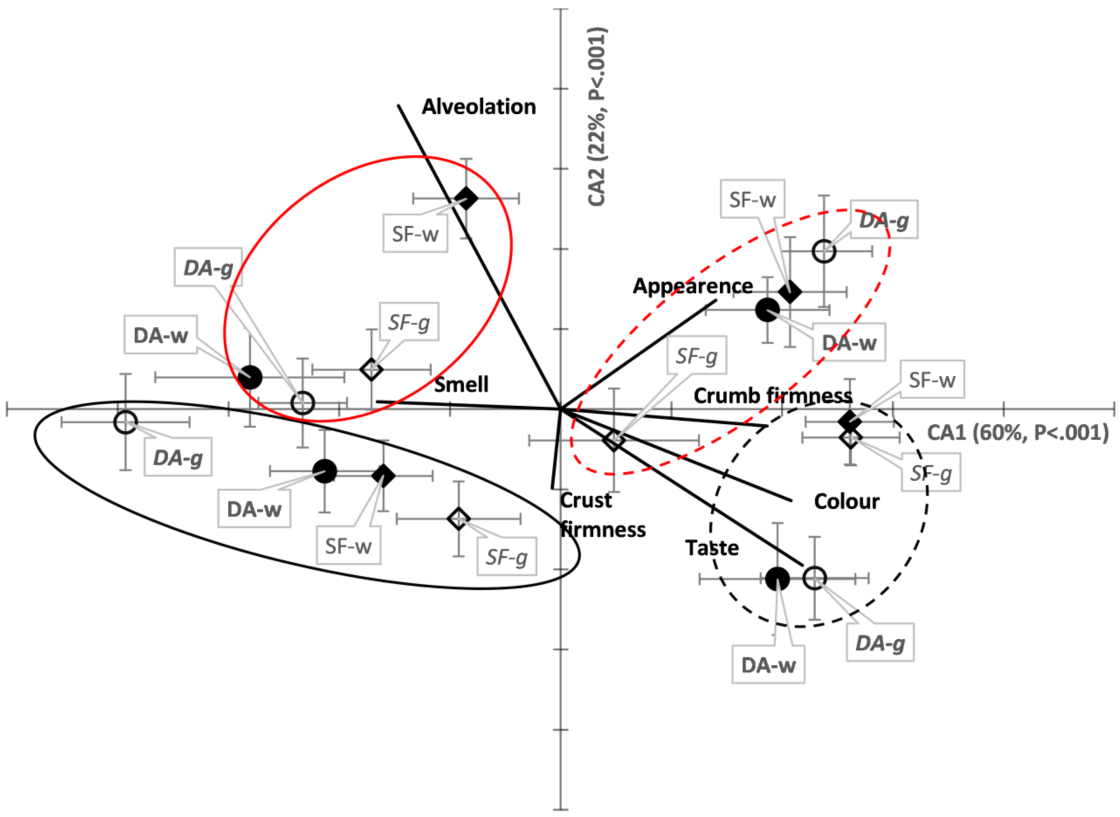
CA2 (22%, P<.001)
CA1 (60%, P<.001)
Alveolation
Appearence
Smell
Crumb firmness
Crust firmness
Colour
Taste
SF-w
DA-g
SF-g
DA-w
DA-g
SF-w
DA-w
SF-g
SF-w
SF-g
DA-g
DA-w
SF-w
SF-g
DA-w
DA-g

Legend.

Supplementary Material Figure S3. Spider plot of sensory attribute's score for the durum wheat breads. G, durum wheat genotype: old genotype ‘Dauno III’ (DA) *vs.* modern genotype ‘Sfinge’ (SF); M, milling product: wholemeal (WHM) *vs.* semolina (SEM); L, leavening agent: brewing yeast (BY) or sourdough (SD); B, baking mode: gas-fired oven (g) or wood-fired oven (w).

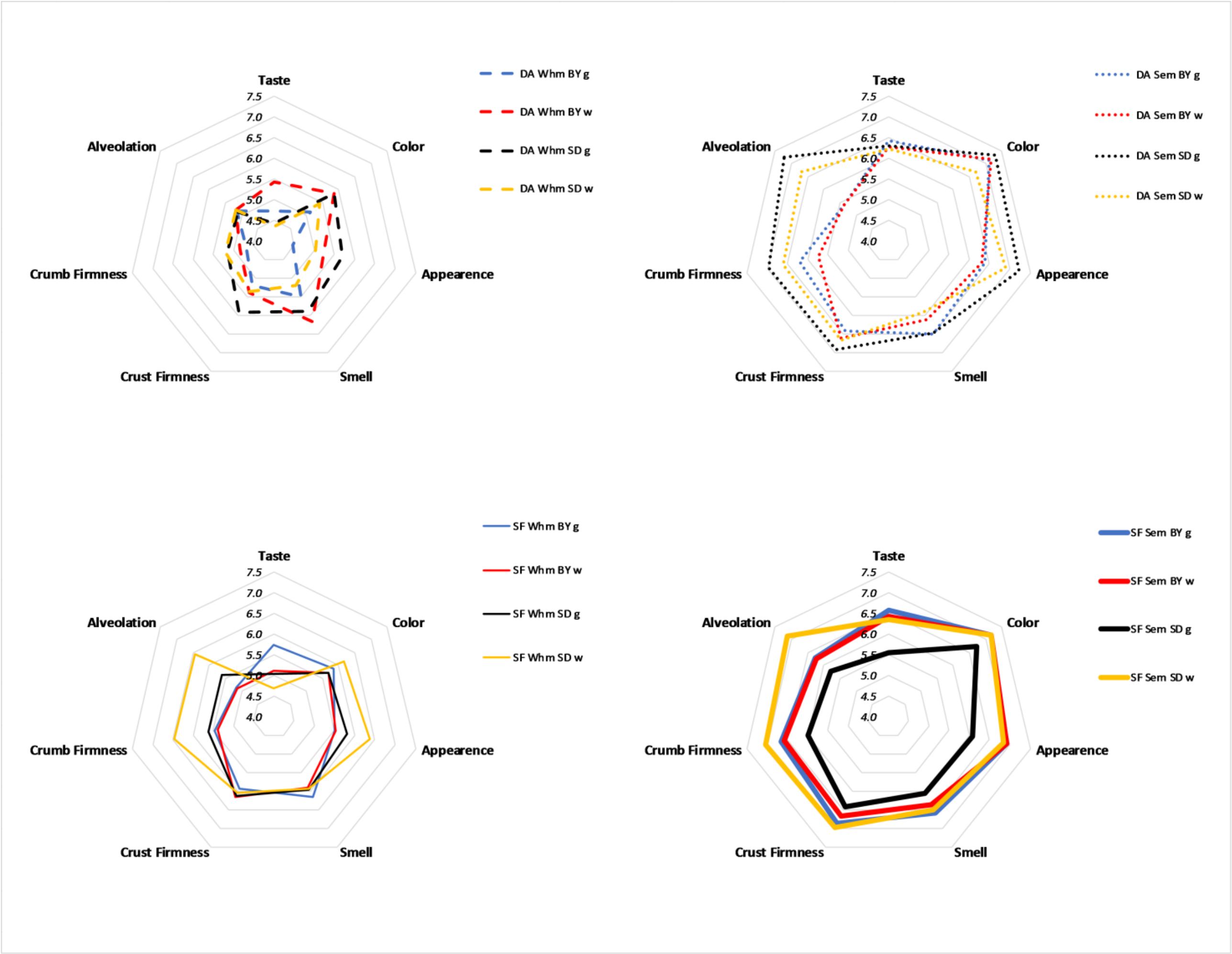
Taste
Color
Appearence
Smell
Crust Firmness
Crumb Firmness
Alveolation
7.5
7.0
6.5
6.0
5.5
5.0
4.5
4.0
DA Whm BY g
DA Whm BY w
DA Whm SD g
DA Whm SD w
DA Sem BY g
DA Sem BY w
DA Sem SD g
DA Sem SD w
SF Whm BY g
SF Whm BY w
SF Whm SD g
SF Whm SD w
SF Sem BY g
SF Sem BY w
SF Sem SD g
SF Sem SD w

Legend.

Table S1. Results of the analysis of variance for contribution of each VOC class to the total VOCs in the breads and milling products studied. MAT is the material (i.e. milling product itself or bread); G, durum wheat genotype (old genotype 'Dauno III' or modern genotype 'Sfinge'); M, milling product (wholemeal or semolina); L, leavening agent (brewing yeast or sourdough); B, baking mode (gas-fired oven or wood-fired oven).

	Alcohols				Aldehydes				Terpenes				Oxime-, methoxy-phenyl				Ketones				Ethylbenzene				2-pentylfuran			
	DFnum[a]	DFden[b]	F[c]	*p*	DFnum	DFden	F	*p*	DFnum	DFden	F	*p*	DFnum	DFden	F	*p*	DFnum	DFden	F	*p*	DFnum	DFden	F	*p*	DFnum	DFden	F	*p*
**MAT**	1	39	615.89	<.0001	1	40	120.12	<.0001	1	37	732.66	<.0001	1	1	173.30	0.0483	n.a.[e]	n.a.	n.a.	n.a.	n.a.	n.a.	n.a.	n.a.	n.a.	n.a.	n.a.	n.a.
**MAT×G**	2	39	33.21	<.0001	2	40	5.61	0.0071	2	37	8.53	0.0009	2	1	20.40	0.1547	1	32	0.75	0.3919	1	32	19.76	<.0001	1	31	3.21	0.0831
**MAT×M**	2	39	63.18	<.0001	2	40	45.48	<.0001	2	37	14.29	<.0001	1[d]	1	16.09	0.1556	1	32	80.30	<.0001	1	32	0.33	0.5695	1	31	197.31	<.0001
**MAT×G×M**	2	39	4.70	0.0149	2	40	1.51	0.2325	1	37	0.49	0.4898	1	1	3.52	0.3117	1	32	87.76	<.0001	1	32	11.09	0.0022	1	31	3.53	0.0698
**L (MAT)**	1	39	27.63	<.0001	1	40	0.36	0.5508	1	37	6.77	0.0132	1	1	70.36	0.0755	1	32	29.33	<.0001	1	32	68.78	<.0001	1	31	11.99	0.0016
**B (MAT)**	1	39	41.56	<.0001	1	40	17.79	0.0001	1	37	8.41	0.0063	1	1	4.10	0.2920	1	32	0.22	0.6446	1	32	8.55	0.0063	1	31	23.88	<.0001
**L×B (MAT)**	1	39	17.08	0.0002	1	40	0.51	0.4788	1	37	0.21	0.6524	1	1	0.12	0.7873	1	32	0.95	0.3359	1	32	1.67	0.2061	1	31	2.33	0.1374
**G×L (MAT)**	1	39	20.19	<.0001	1	40	0.21	0.6486	1	37	165.10	<.0001	1	1	40.15	0.0997	1	32	48.06	<.0001	1	32	0.28	0.6026	1	31	18.50	0.0002
**G×B (MAT)**	1	39	6.01	0.0189	1	40	0.97	0.3297	1	37	25.08	<.0001	1	1	13.08	0.1717	1	32	0.33	0.5695	1	32	11.37	0.0020	1	31	0.44	0.5138
**M×L (MAT)**	1	39	17.30	0.0002	1	40	70.14	<.0001	1	37	46.62	<.0001	1	1	72.13	0.0746	1	32	2.76	0.1067	1	32	150.34	<.0001	1	31	2.07	0.1598
**M×B (MAT)**	1	39	15.23	0.0004	1	40	0.85	0.3634	1	37	31.45	<.0001	1	1	106.25	0.0616	1	32	0.02	0.8811	1	32	104.22	<.0001	1	31	41.01	<.0001
**G×M×L (MAT)**	1	39	0.40	0.5320	1	40	0.42	0.5220	1	37	1.72	0.1975	1	1	4.02	0.2946	1	32	1.12	0.2970	1	32	0.02	0.8868	1	31	0.30	0.5903
**G×M×B (MAT)**	1	39	0.82	0.3705	1	40	0.00	0.9755	1	37	1.15	0.2898	1	1	0.12	0.7871	1	32	4.61	0.0394	1	32	1.51	0.2278	1	31	1.19	0.2837
**G×L×B (MAT)**	1	39	1.08	0.3051	1	40	3.04	0.0891	1	37	0.08	0.7825	1	1	0.58	0.5850	1	32	20.30	<.0001	1	32	0.28	0.6033	1	31	0.71	0.4049
**M×L×B (MAT)**	1	39	18.35	0.0001	1	40	2.15	0.1503	1	37	1.98	0.1682	1	1	0.68	0.5606	1	32	11.87	0.0016	1	32	5.33	0.0276	1	31	0.02	0.9017
**G×M×L×B (MAT)**	1	39	1.60	0.2138	1	40	0.18	0.6753	1	37	1.31	0.2605	1	1	0.00	0.9825	1	32	2.37	0.1333	1	32	0.02	0.8999	1	31	2.88	0.0998

Legend.

Table S2. Sensory perception values (±S.E.) and results of the analysis of variance (the general linear mixed model). G, durum wheat genotype (old genotype 'Dauno III' or modern genotype 'Sfinge'); M, milling product (wholemeal or semolina); L, leavening agent (brewing yeast or sourdough); B, baking mode (gas-fired oven or wood-fired oven). Values in bold are at $p<0.05$.

				Taste	Color	Appearence	Smell	Crust Cons.	Crumb Cons.	Alveolation
Dauno III	Wholemeal	BY	g	4.7 ± 0.28	5.1 ± 0.42	4.5 ± 0.33	5.5 ± 0.41	5.2 ± 0.38	4.7 ± 0.28	5.2 ± 0.30
			w	5.4 ± 0.30	5.8 ± 0.27	5.2 ± 0.17	6.2 ± 0.27	5.4 ± 0.29	4.8 ± 0.31	5.2 ± 0.30
		SD	g	4.4 ± 0.29	5.8 ± 0.28	5.7 ± 0.16	5.9 ± 0.28	5.9 ± 0.19	5.2 ± 0.39	5.1 ± 0.25
			w	4.3 ± 0.37	5.4 ± 0.34	5.0 ± 0.34	5.2 ± 0.35	5.3 ± 0.42	5.2 ± 0.41	5.2 ± 0.36
	Semolina	BY	g	6.4 ± 0.41	7.1 ± 0.18	6.4 ± 0.28	6.5 ± 0.30	6.4 ± 0.26	6.2 ± 0.29	5.4 ± 0.32
			w	6.3 ± 0.21	7.2 ± 0.23	6.3 ± 0.32	6.1 ± 0.36	6.6 ± 0.25	5.7 ± 0.31	5.4 ± 0.26
		SD	g	6.3 ± 0.21	7.3 ± 0.17	7.2 ± 0.22	6.5 ± 0.38	6.9 ± 0.14	7.0 ± 0.20	7.2 ± 0.26
			w	6.2 ± 0.31	6.7 ± 0.30	6.9 ± 0.19	5.9 ± 0.45	6.7 ± 0.23	6.6 ± 0.22	6.7 ± 0.22
Sfinge	Wholemeal	BY	g	5.7 ± 0.31	5.8 ± 0.26	5.5 ± 0.17	6.2 ± 0.26	5.9 ± 0.20	5.5 ± 0.27	5.2 ± 0.22
			w	5.1 ± 0.28	5.7 ± 0.29	5.5 ± 0.19	5.9 ± 0.32	6.2 ± 0.15	5.4 ± 0.21	5.1 ± 0.20
		SD	g	5.0 ± 0.24	5.7 ± 0.36	5.8 ± 0.17	6.0 ± 0.33	6.1 ± 0.17	5.6 ± 0.20	5.6 ± 0.18
			w	4.7 ± 0.26	6.2 ± 0.32	6.4 ± 0.27	5.9 ± 0.38	6.0 ± 0.34	6.5 ± 0.24	6.4 ± 0.27
	Semolina	BY	g	6.6 ± 0.19	7.2 ± 0.13	6.9 ± 0.19	6.6 ± 0.25	6.8 ± 0.24	6.7 ± 0.20	6.3 ± 0.22
			w	6.4 ± 0.24	7.2 ± 0.18	6.9 ± 0.20	6.3 ± 0.40	6.7 ± 0.21	6.6 ± 0.20	6.2 ± 0.28
		SD	g	5.5 ± 0.32	6.7 ± 0.28	6.1 ± 0.39	6.0 ± 0.35	6.4 ± 0.31	6.0 ± 0.38	5.8 ± 0.44
			w	6.3 ± 0.27	7.2 ± 0.18	6.8 ± 0.30	6.5 ± 0.25	7.0 ± 0.17	7.0 ± 0.16	7.1 ± 0.31

	F	*p*	F	*p*	F	*p*	F	*p*	F	*p*	F	*p*	F	*p*
Genotype (G)	1.33	*0.261*	1.09	*0.306*	**6.08**	***0.021***	2.36	*0.137*	**6.57**	***0.017***	**16.93**	***<.001***	4.20	*0.052*
Milling (M)	**95.0**	***<.001***	**176.7**	***<.001***	**122.1**	***<.001***	**12.5**	***0.001***	**73.4**	***<.001***	**93.8**	***<.001***	**49.3**	***<.001***
G×M	3.37	*0.068*	2.28	*0.133*	**10.04**	***0.002***	0.46	*0.498*	**5.96**	***0.016***	**6.01**	***0.015***	0.90	*0.344*
Leavening agent (L)	**11.98**	***0.001***	0.01	*0.925*	**8.99**	***0.003***	1.57	*0.212*	1.89	*0.171*	**14.30**	***<.001***	**27.06**	***<.001***
G×L	0.40	*0.526*	0.04	*0.851*	**6.17**	***0.014***	0.06	*0.803*	2.15	*0.145*	2.36	*0.126*	0.90	*0.344*
M×L	1.28	*0.260*	2.57	*0.111*	3.55	*0.061*	0.01	*0.915*	0.16	*0.690*	0.39	*0.534*	3.18	*0.076*
G×M×L	1.28	*0.260*	0.04	*0.851*	**7.51**	***0.007***	0.29	*0.592*	0.00	*0.965*	**6.01**	***0.015***	**20.42**	***<.001***
Baking mode (B)	0.00	*0.944*	0.32	*0.572*	1.44	*0.232*	0.93	*0.336*	0.00	*0.965*	1.45	*0.230*	2.67	*0.104*
G×B	0.40	*0.526*	1.50	*0.222*	3.55	*0.061*	0.68	*0.412*	1.23	*0.269*	**6.43**	***0.012***	**6.09**	***0.015***
M×B	0.50	*0.481*	0.89	*0.347*	0.18	*0.669*	0.10	*0.748*	0.33	*0.565*	0.76	*0.384*	0.01	*0.909*
G×M×B	**5.11**	***0.025***	1.50	*0.222*	0.59	*0.442*	1.75	*0.188*	0.02	*0.894*	1.66	*0.199*	1.39	*0.241*
L×B	0.24	*0.622*	0.89	*0.347*	0.18	*0.669*	0.29	*0.592*	0.87	*0.353*	**4.85**	***0.029***	2.92	*0.089*
G×L×B	3.12	*0.079*	**12.17**	***0.001***	**11.16**	***0.001***	**5.08**	***0.026***	3.64	*0.058*	**4.85**	***0.029***	**7.27**	***0.008***
M×L×B	2.00	*0.160*	0.14	*0.707*	2.38	*0.125*	2.36	*0.126*	2.41	*0.122*	0.21	*0.648*	0.00	*0.970*
G×M×L×B	0.02	*0.888*	0.72	*0.397*	1.06	*0.306*	0.46	*0.498*	0.71	*0.400*	0.00	*0.967*	1.21	*0.272*
